# Transcatheter aortic valve replacement planning with cardiac computed tomography in quadricuspid aortic valve stenosis: a case series

**DOI:** 10.1093/ehjcr/ytae079

**Published:** 2024-02-08

**Authors:** Heberto Aquino-Bruno, Roberto Muratalla-González, Juan F Garcia-Garcia, José L Triano-Doroteo, Kevin Felix Rivera, Gerardo Carreon Balcarcel, Marisol Navarrete-Osuna

**Affiliations:** Interventional Cardiology Service, Centro Medico Nacional 20 de Noviembre, Av. Felix Cuevas #540, Col. Del Valle Del. Benito Juarez, Mexico City 03100, Mexico; Interventional Cardiology Service, Centro Medico Nacional 20 de Noviembre, Av. Felix Cuevas #540, Col. Del Valle Del. Benito Juarez, Mexico City 03100, Mexico; Interventional Cardiology Service, Centro Medico Nacional 20 de Noviembre, Av. Felix Cuevas #540, Col. Del Valle Del. Benito Juarez, Mexico City 03100, Mexico; Interventional Cardiology Service, Hospital Regional Culiacan ISSSTE, Sinaloa, Mexico; Interventional Cardiology Service, Hospital Regional Culiacan ISSSTE, Sinaloa, Mexico; Interventional Cardiology Service, Hospital Regional Veracruz ISSSTE, Veracruz, Mexico; Ecocardiography Cardiology Service, Hospital Regional Veracruz ISSSTE, Veracruz, Mexico

**Keywords:** Case series, Quadricuspid aortic valve, TAVR, CT cardiac

## Abstract

**Background:**

The presence of severe aortic stenosis in quadricuspid aortic valve (QAV) is an extremely rare combination, and it is unknown whether transcatheter aortic valve replacement (TAVR) is a safe option due to the low incidence.

**Case summary:**

We present two patients diagnosed with severe aortic stenosis with QAV morphology type 1 (Nakamura classification). All patients presented to our hospital for evaluation because of worsening functional class, dyspnoea, or syncope. During tomographic planning, the aortic annulus was measured at the level of the deepest sinus for the selection of the number of devices. Due to the presence of four cusps, the smallest cusp was excluded, and three sinuses were virtualized for placement of the pigtail catheter during the procedure. Without complications, a 23 mm Edwards SAPIEN 3 was deployed through the femoral artery in both patients. Control aortography showed no valve leakage or regurgitation.

**Discussion:**

In patients with QAV and aortic stenosis undergoing TAVR, similar to the tricuspid valve, tomographic planning can be used to ensure the success of the procedure. However, unlike the tricuspid valve, where the selection of the device number is based on the measurements of the aortic annulus at the level of the non-coronary sinus, in these QAV cases, we perform the measurements at the level of the deepest aortic sinus (right coronary sinus).

**Learning points**

- Pre-operative measurements and strategy planning are extremely important for TAVR procedures. Virtual simulation of three sinuses, similar to patients with tricuspid valves, is an adequate measure for valve alignment during the procedure.
- Correct measurement of the annulus is very important in patients with quadricuspid valves, and annular measurement can be selected at the level of the deepest sinus.
- Balloon-expandable TAVR is safe and accessible in patients with QAV.

## Introduction

Quadricuspid aortic valve (QAV) is a rare congenital heart defect with an estimated incidence of <0.05%. It is usually associated with progressive aortic regurgitation (AR), whereas aortic stenosis (AS) is uncommon.^1^

Surgical replacement or repair has historically been the standard treatment for QAV with severe AS or AR, but with the advent of TAVR, treatment is under debate.^2,3^ The differences between QAV and tricuspid aortic valve (TAV) in the shape of the aortic sinus, the location of the coronary orifices, the length of a single valve leaflet, and the distribution of calcification make the anatomy challenging.

We present two cases of AS in QAV outside of surgical treatment, which were treated by TAVR where tomographic planning was crucial for the success of the procedure.

## Summary figure

**Figure:**
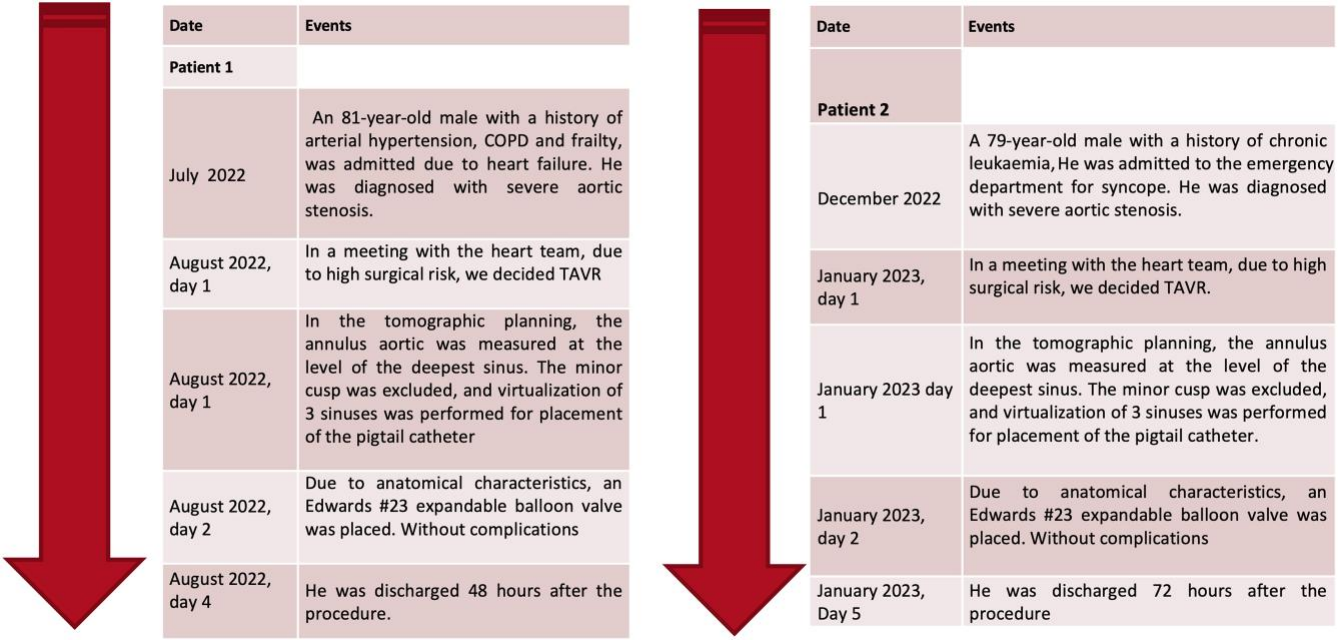


## Case 1

An 81-year-old man with a history of arterial hypertension, COPD, and frailty was admitted to our hospital due to progressive dyspnoea (*Table 1*). Upon admission, vital signs were as follows: blood pressure of 118/63 mmHg, heart rate 78 b.p.m., respiratory rate 19 breaths per minute, and pulse oximetry 92% at ambient air. Transthoracic echocardiography showed severe AS with a peak velocity of 4.3 m/s, maximum gradient of 74 mmHg, mean pressure gradient of 46 mmHg, and aortic valve area of 0.63 cm^2^ (index of 0.4 cm^2^/m^2^) with moderate regurgitation, LVEF of 55% with LV hypertrophy. Coronary angiography was reported without significant lesions. Due to high surgical risk, the heart team determined that he was a suitable candidate for TAVR.

**Table 1:** Pre-operative clinical characteristics

Patients	Sex	Age (years)	Functional status of aortic valve	Clinical presentation	STS score	NYHA FC	Comorbidities
Patient 1	Male	81	Severe AS/moderate AI	Dyspnoea	8.1	III	Systemic arterial hypertension, COPD and frailty
Patient 2	Male	79	Severe AS/mild AI	Syncope	9.2	III	Chronic lymphocytic leukemia

AI, aortic insufficiency; AS, aortic stenosis; COPD, chronic obstructive pulmonary disease.

**Figure 1:**
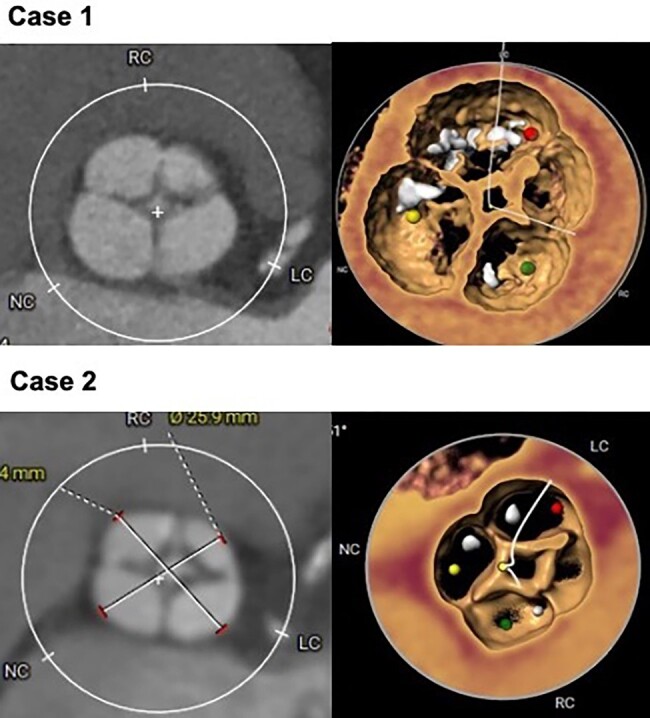
Quadricuspid aortic valve, with an accessory cusp between the right and left coronary sinuses, type 1 of the Nakamura classification. 3D reconstruction, with the simulation of the three sinuses.

**Figure 2:**
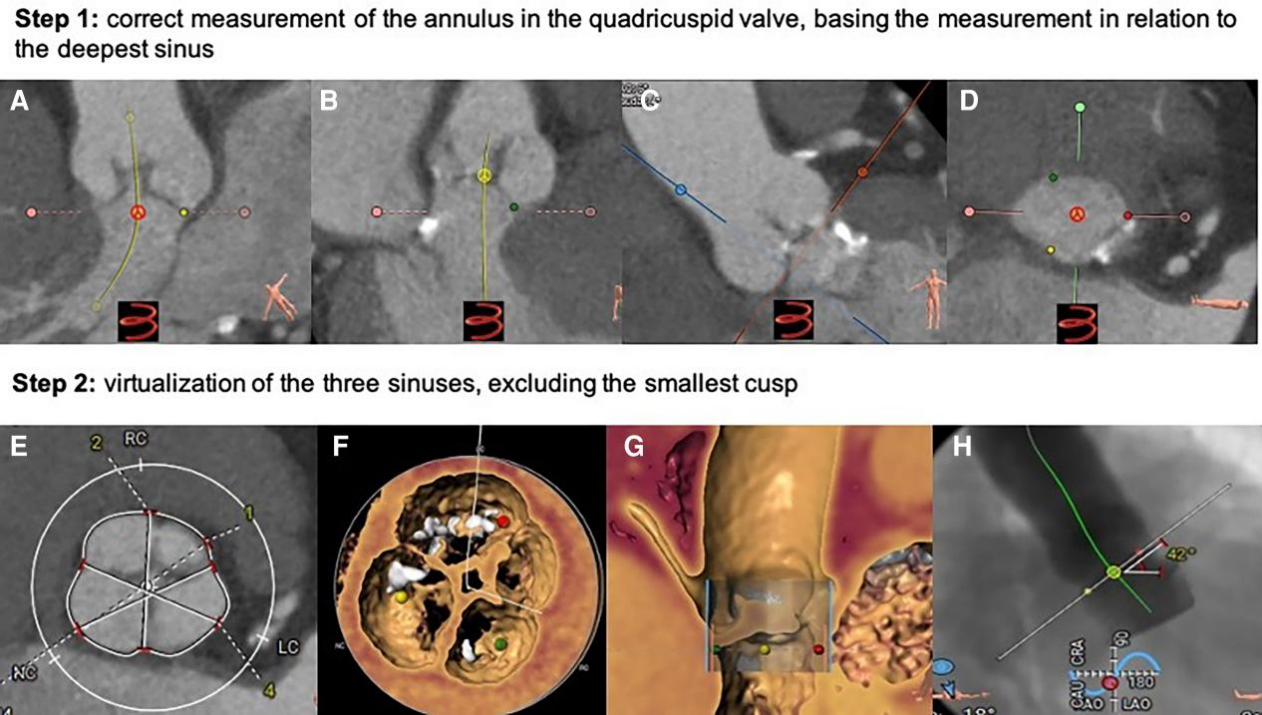
Planning tomographic: (*A*) Measurement of the depth of the non-coronary sinus (yellow point). (*B*) Depth of the right coronary sinus (green point). (*C*) Depth of the left coronary sinus (red point). (*D*) Measurement of the annulus ring based on the deepest sinus (right coronary sinus, green dot). (*E* and *F*) Simulation of the three sinuses in QAV and exclusion of the accessory coronary sinus. (*G* and *H*) Cusp overlap and working position.

**Figure 3:**
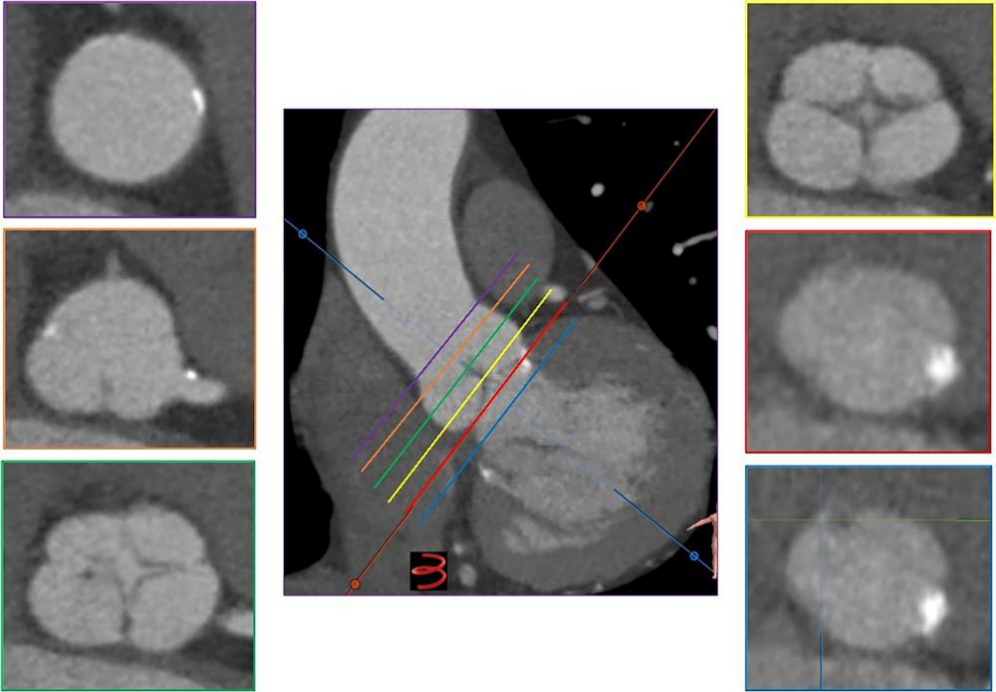
Measurement of virtual landing zone on computed tomography: Descending from the aortic root (purple line), sinutubular junction (orange line), and cusps (green and yellow lines) to the left ventricular outflow track (line blue), the hinge point of each individual aortic valve cusp is identified to establish the plane of the annulus (red line). Virtual ring of aortic annulus was selected at the plane of the circumferential ring at the basal attachment points of the aortic cusps (red line).

Cardiac computed tomography (CT) showed a QAV, with an accessory cusp between the right and left coronary sinuses, type 1 of the Nakamura classification (*Figure 1*). Secondary to the presence of four cusps, the minor cusp was excluded, and 3D reconstruction, with the simulation of three sinuses, was performed for the placement of the pigtail catheter (*Figures 1* and *2*). Subsequently, similar to a tricuspid valve, the measurement of the aortic annulus was performed to select the size of the prosthesis, and the landing zone was evaluated. Calcium was observed extending from the base of the left coronary cusp into the LV outflow tract. The annulus was measured at the level of the deepest sinus that was the right coronary sinus (annulus area 369.7 mm^2^, annulus perimeter 69.7; *Figure 3* and *Table 2*).

**Table 2:** Pre-operative computed tomography measurements and procedural characteristics

Patients	Quadricuspid type (Nakamura classification)	Annulus perimeter (mm)	Annulus area (mm^2^)	Sinus diameter (mm)	STJ diameter (mm)	LCA height (mm)	RCA height (mm)	Calcification
Patient 1	I	69.7	369.7	26	25.8	15.8	16.5	Mild
Patient 2	I	65.9	325.4	26.4	23.3	11.5	12.4	Mild

LCA, left coronary artery; RCA, right coronary artery; STJ, sinutubular junction.

**Figure 4:**
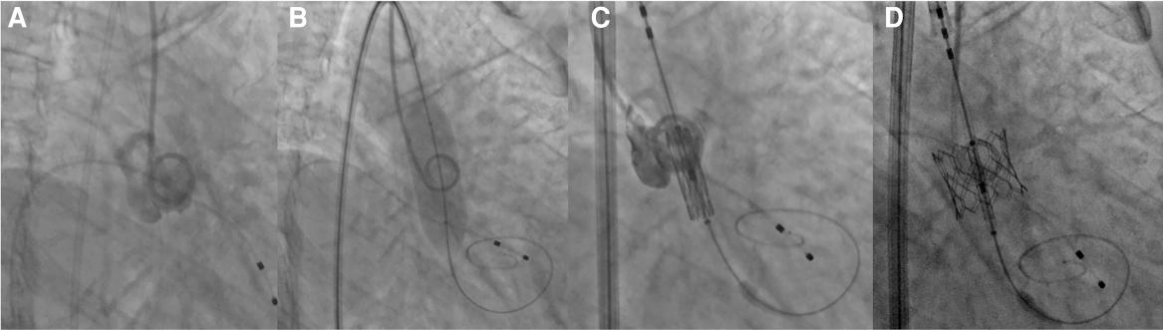
(*A*) Pigtail placement in the right coronary sinus, centralization of the same sinus was performed, with the purpose of locating the non-coronary sinus on the left of the image and the left coronary sinus on the right side during the injection of the contrast medium. (*B*) Pre-dilatation with #18 balloon. (*C*) Position of the valve at the depth level of the right coronary sinus. (*D*) Valve deployment without complications.

With a bilateral femoral arterial approach and under sedation, the pigtail placement in the right coronary sinus, centralization of the same sinus was performed, with the purpose of locating the non-coronary sinus on the left of the image and the left coronary sinus on the right side during the injection of the contrast medium. With pacing at 180 b.p.m., we did dilatation with #18 balloon, and subsequently, an Edwards SAPIEN 3 valve #23 was placed (*Figure 4*; see Supplementary material online, *Videos S1–S4*). Control aortography showed the lack of paravalvular leak or valvular regurgitation. The control echocardiogram reported a mean gradient of 7 mmHg and a velocity of 1.7 m/s. He was discharged 48 h after the procedure (*Table 3*).

**Table 3:** Procedural characteristics

Patients	Approach	TAVR devices used	Pre-dilatation	Post-dilatation	Procedural success	Procedural complications	Length of hospital stay (days)
Patient 1	Transfemoral	Edwards SAPIEN 3 23mm	Yes	No	Yes	No	2
Patient 2	Transfemoral	Edwards SAPIEN 3 23mm	Yes	No	Yes	No	3

TAVR, transcatheter aortic valve replacement.

## Case 2

A 79-year-old male patient with a history of chronic lymphocytic leukemia was admitted to the emergency department for syncope (*Table 1*). Vitals at admission were as follows: blood pressure of 122/71 mmHg, heart rate 88 b.p.m., respiratory rate 17 breaths per minute, and pulse oximetry 95% at ambient air. Transthoracic echocardiography showed severe AS with a peak velocity of 4.1 m/s, a maximum gradient of 72 mmHg, a mean pressure gradient of 42 mmHg, an aortic valve area of 0.7 cm^2^ (index 0.3 cm^2^/m^2^), and LVEF of 55% with concentric ventricular hypertrophy.

Computed tomography confirmed the presence of a type 1 QAV with an accessory cusp between the right and left coronary sinuses. Due to the high surgical risk, he was accepted for TAVR. Similar to the previous patient, a tomographic analysis and planning of the procedure were done, the minor cusp was excluded, and the three-sinus virtualization was performed for pigtail catheter placement. Cardiac CT measure revealed an annulus area of 325.4 mm^2^ and an annulus perimeter of 65.9 mm (*Table 1*).

Bilateral femoral access was obtained using ultrasound imaging. The aortic valve was crossed with a 0.0035 inch straight-tip wire that was later exchanged for a 0.0035 inch Safari 2 stiff wire using an Amplatz left (AL) catheter. Under sedation, with pacing at 180 b.p.m., we did dilatation with #18 balloon, and after pre-dilatation, an Edwards SAPIEN 3 valve #23 was placed. Control aortography showed the lack of paravalvular leak or valvular regurgitation. The control echocardiogram showed a mean gradient of 5 mmHg and a velocity of 1.5 m/s. The patient was discharged 72 h after the procedure (*Table 3*).

At 6-month follow-up of both patients, post-TAVR haemodynamic behaviour has been satisfactory; no conduction alteration was observed post-procedure or during follow-up.

## Discussion

Quadricuspid aortic valve is a rare congenital heart defect with an estimated incidence of <0.05%.^1^ The haemodynamic abnormality is related to valve subtypes according to the Hurwitz and Roberts classification; in the most extensive study of QAVs (49 patients), most were associated with AR of any degree, present in 45 patients (90%), of whom 13 (26%) had moderate or severe AR. Only four patients (8%) had AS, which was mild in all four.^4^

Under normal conditions, the aortic valve has three leaflets or cusps, named right coronary, left coronary, and non-coronary, named for their relationship with the coronary arteries. These anatomical characteristics have implications during the procedure planning for TAVR for valve alignment (cusp overlap), particularly the non-coronary sinus.^5^

Among its anatomical variants, there may be significant differences between the QAV and the TAV in the shape of the aortic sinus, different aortic sinus morphologies, and the distribution of calcification. In addition, the distribution of coronary ostia in the QAV differs from that of the TAV according to the shape of the aortic sinus.^3,5^ These anatomical features take into account the interaction between the transcatheter heart valve (THV) frame and the aortic valve complex, which may have important implications for THV expansion and orientation, as well as post-procedural outcomes.

Pre-operative measurements and strategy planning for TAVR procedures are essential. The current gold standard method for performing valve measurements and pre-procedural TAVR planning is cardiac CT with an appropriate TAVR protocol.^5^ The approach, type and size of THV, and procedural risk prediction are all based on pre-operative measurements and tomographic assessment. The Hurwitz and Roberts classification is based on the relative size of the supernumerary cusp; however, this classification has limited utility for surgical guidance.^4^ Nakamura *et al.*^6^ developed a simplified classification by focusing on the location of the supernumerary cusp. Although it has been reported that the location of the supernumerary cusp does not influence clinical outcomes, this classification may be important during tomographic evaluation to exclude the smallest cusp and perform the three-sinus virtualization.

There is no systematization for tomographic planning in a patient with QAV, due to the low reported incidence; however, patients with bicuspid aortic valves (BAVs), who have undergone TAVR, have shown favourable results with tomographic planning with virtual realization of three sinuses.^5,7^ It should be taken into account that the anatomy of patients with BAV presents a completely different anatomy from that of TAV, due to the presence of raphes and an eccentric aortic orifice; on the contrary, QAVs have a similar functionality to TAV, with a central aortic orifice without raphes.

In tricuspid AS, the virtual basal ring is the tightest part of the aortic root, where the THV will anchor and seal.^8^ Hence, this virtual structure, bounded by three anatomic hinge points at the nadir of each of the aortic cusp insertions, represents the appropriate reference plane for prosthesis sizing and implantation height. In BAV, the anatomic definition of the virtual basal ring may be more difficult.^9^ We observe that unlike bicuspid valves, the anatomical characteristics of the landing zone (aortic annulus) in QAV are similar to a tricuspid valve (*Figure 3*); therefore, in patients with QAV and AS undergoing TAVR, it is possible to opt for tomographic planning of the procedure, similar to a patient with a tricuspid valve, for the success of the procedure. However, unlike the tricuspid valve, where the selection of the device number is based on the measurements of the aortic annulus at the level of the non-coronary sinus, in these cases, we perform the measurements at the level of the deepest aortic sinus (right coronary sinus).

The introduction of the new-generation THVs has improved the device success rate in BAV similarly to TAV.^10^ The TAV-like results could be expected in QAV due to the favourable anatomy for TAVR. There are still no large studies on the results of TAVR in QAV, as it is a rare combination; however, according to the few reported cases treated with TAVR, the anatomy seems to be accessible, with favourable results provided that proper planning is done. According to the literature review, balloon-expandable valves may be an adequate and safe option in this type of anatomy.

According to the two procedures performed with these anatomical characteristics, we consider an accessible anatomy for TAVR. During both procedures, the anatomical behaviour was accessible both in pre-dilatation and during valve deployment.

## Supplementary Material

ytae079_Supplementary_Data

## Data Availability

The data used and/or analysed during the current study are available from the corresponding author upon reasonable request.

## Lead author biography

Dr Heberto Aquino-Bruno is a clinical cardiologist who graduated from the ISSSTE November 20th National Medical Center. He is currently doing a fellowship in interventional cardiology and pursuing a master’s degree in health sciences at the ISSSTE November 20th National Medical Center. His main area of interest is coronary and structural intervention. He has made several publications on TAVR.
